# The Golden Retr iever Lifetime Study: Assessing factors associated with owner compliance after the first year of enrollment

**DOI:** 10.1111/jvim.15921

**Published:** 2020-11-16

**Authors:** Audrey Ruple, Melissa Jones, Missy Simpson, Rodney Page

**Affiliations:** ^1^ Department of Public Health College of Health and Human Sciences, Purdue University West Lafayette Indiana USA; ^2^ Department of Clinical Sciences College of Veterinary Medicine, Purdue University West Lafayette Indiana USA; ^3^ Morris Animal Foundation Denver Colorado USA; ^4^ Flint Animal Cancer Center College of Veterinary Medicine and Biomedical Sciences, Colorado State University Fort Collins Colorado USA

**Keywords:** cohort, longitudinal, participant selection, study compliance

## Abstract

**Background:**

The Golden Retriever Lifetime Study (GRLS) is one of the largest canine cohort studies undertaken in the United States to date. This study design allows for evaluation of multiple exposures and outcomes throughout the lifetime of each dog, but relies on participants to comply with study requirements over a long period of time. Failure to do so can lead to biased reporting of results.

**Objectives:**

To examine factors associated with dog owner compliance for GRLS.

**Animals:**

Golden Retrievers (n = 3044) whose owners elected to participate in GRLS.

**Methods:**

Prospective, cohort study. A logistic regression model was constructed to examine associations between data collected at the time of initial enrollment in GRLS and the outcome of failure to fulfill all study obligations at the end of the first year after enrollment in GRLS.

**Results:**

There were 192 (6.3%) owners who did not comply with study requirements 1 year after enrollment. Owners of dogs without a record of vaccination had nearly 4 times higher odds (adjusted OR: 3.7, 95% CI: 1.5, 9.2) of being noncompliant than owners of vaccinated dogs and owners of dogs that slept in the garage had nearly 6 times higher odds (adjusted OR: 5.7, 95% CI: 1.9, 17.0) of being noncompliant than owners of dogs that slept in their bedroom.

**Conclusions and Clinical Importance:**

Survey questions about a dog's sleeping location at night and vaccination status are important indicators of an owner's odds of compliance in a prospective study. Use of similar questions during enrollment in cohort studies might help to predict owner compliance that can aid in subject selection.

**Abbreviations**
AAHA: American Animal Hospital Association
C‐BARQ: Canine Behavioral Assessment and Research Questionnaire
GRLS: Golden Retriever Lifetime Study
OR: odds ratio
95% CI: 95% confidence interval

## 1 INTRODUCTION

Prospective cohort studies like the Framingham Heart Study and the Nurses' Health Study have proven to be valuable for collecting a large amount of epidemiological data about human populations over an extended period of time.^1^, ^2^, ^3^ Longitudinal cohort studies are less commonly utilized in veterinary medicine than in human medicine, but the use of this type of study can be valuable for investigating the complicated relationships between genetic and environmental exposures and disease outcomes within a dog population. In fact, genetic variation is reduced within dog breeds, making disease mapping within a single dog breed more efficient as compared to the use of the same technique in humans.^4^, ^5^, ^6^ Furthermore, the canine spontaneous tumor model is ideal for furthering human cancer research because (a) dogs spontaneously develop tumors, which are similar to human tumors; (b) more dogs than people are diagnosed with cancer each year; and (c) dogs age more quickly than people and therefore have an accelerated rate of disease progression.^4^, ^5^

The Golden Retriever Lifetime Study (GRLS), predicted to be a 15‐year cohort study of more than 3000 Golden Retrievers, is currently being conducted by Morris Animal Foundation.^7^ The primary aim of GRLS is to identify risk factors for, and incidence of, common cancers in Golden Retrievers, but information about many other aspects of health and lifestyle can be evaluated within the framework of GRLS. Similarly, a large amount of health and lifestyle of dogs' data are being collected about Labrador Retrievers in the United Kingdom through Dogslife, a web‐based longitudinal study.^8^ Another large‐scale project currently enrolling participants is the Dog Aging Project, which is collecting health and lifestyle data about dogs of all breeds in the United States.^9^ Studies such as these involve a large investment of resources, so efforts must be implemented to achieve the greatest success possible by recruiting a large subject pool with owners likely to comply with study protocols throughout their dog's lifetime. Recruitment, retention, and compliance, especially with regard to adherence to timelines, have all been challenges for Dogslife.^10^ Without past research on strategies for owner recruitment and selection for large longitudinal studies involving dogs and their owners, it is important to gain a better understanding of what factors affect owner compliance as maximizing compliance will improve both the economics of study resource use and the likelihood of study success through efficient data generation. Therefore, the purpose of this study was to examine factors associated with dog owner compliance at their second study visit (1 year after enrollment in GRLS).

## 2 MATERIALS AND METHODS

### 2.1 Overview

All 3044 dogs enrolled in GRLS were included in this study. A logistic regression model was constructed with the primary endpoint being failure to fulfill research obligations at the end of the first year after enrollment in GRLS. Data collected from the owner at the time of enrollment in GRLS were used to examine associations with the outcome of non‐compliance.

### 2.2 Study design and compliance

Enrollment into GRLS was an owner‐driven process that has been described previously.^7^ Briefly, owners of Golden retriever dogs less than 2 years of age with at least a 2 generation purebred pedigree who were free from known chronic diseases living in the continental United States were recruited through the Morris Animal Foundation website, social media, and word of mouth. Owners were asked to complete a survey and take their dog to a veterinarian at the time of enrollment and each year after enrollment throughout the dog's lifetime. Contact information collected from the owners included their email and mailing addresses as well as their phone number. No direct questions about the owner were included in the survey, rather the survey contained questions about their dog's health and lifestyle (activity level, sleeping habits, etc.). Additionally, veterinarians were asked to complete an annual survey after each routine visit and submit samples collected at the time of the visit, including blood, urine, hair, toenail clippings, and feces to a biorepository for long‐term storage.^7^

**FIGURE 1:**
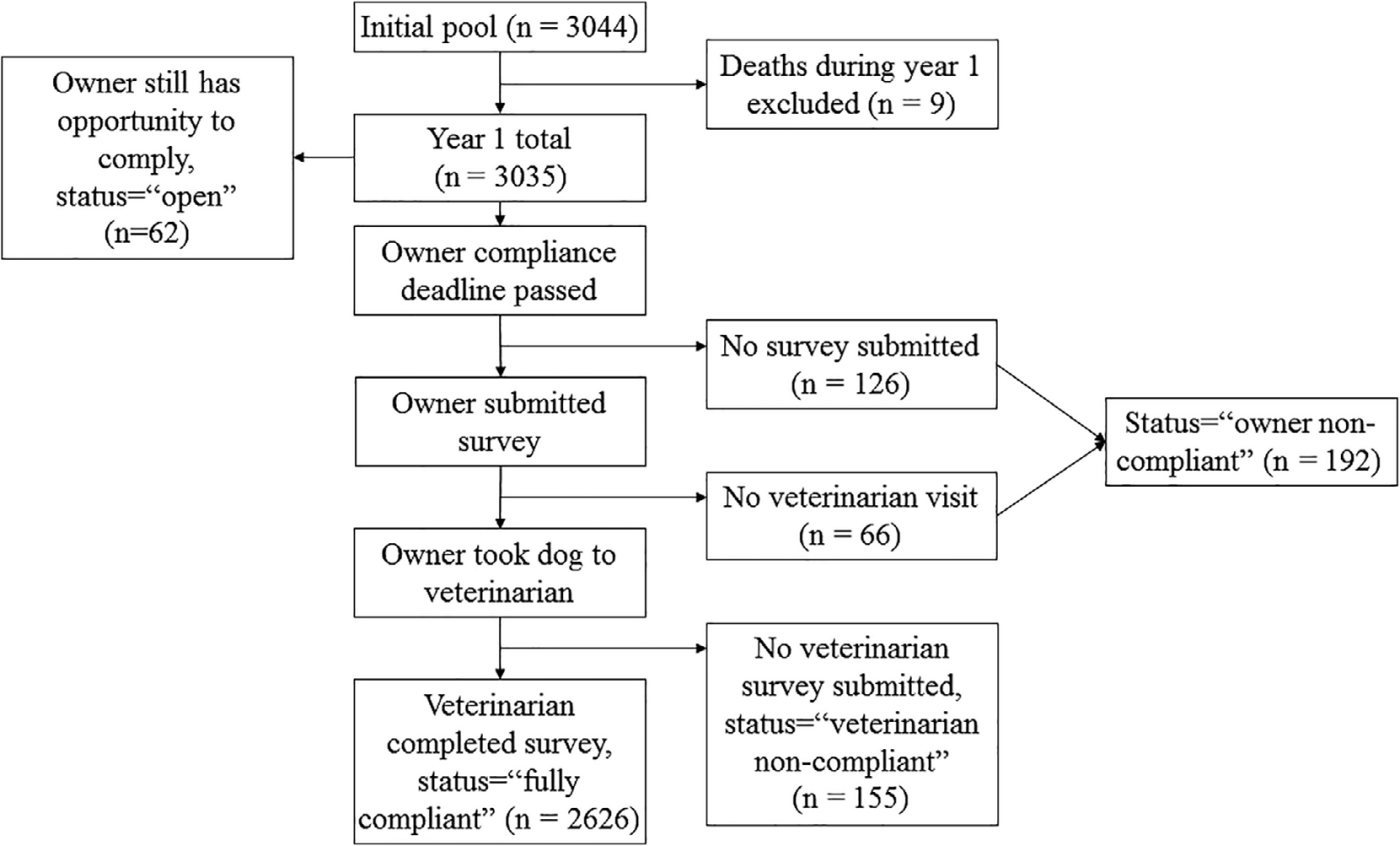
Compliance status of the entire GRLS study population (N = 3035) at the end of study year 1

Owners could begin the annual study processes 90 days before their enrollment anniversary date and had about 6 months after that date to fully complete the requirements. A time‐structured reminder protocol that utilized email, phone, and postcard reminders was employed both in advance of the enrollment anniversary and after the anniversary date if the survey had not been completed. Completion of all study requirements at the initial enrollment visit was a study inclusion criterion. For year 1 after enrollment, dogs were placed into 1 of 4 compliance status categories: open (owner still has opportunity to comply), owner noncompliant (owner did not fulfill any requirements or completed survey only), veterinarian noncompliant (owner was fully compliant, but veterinarian survey had not yet been received), or fully compliant (owner was fully compliant and veterinarian had submitted survey and samples) (Figure 1). For the purposes of this analysis, owners were considered compliant if the information obtained on their dog was complete (fully compliant) or missing the veterinary survey or sample submission (veterinarian noncompliant). Owners were considered noncompliant if they did not complete the survey or if they did not complete a veterinary visit within the required timeframe (owner noncompliant). Owners in the open category, still within the timeframe to be considered compliant should they fulfill study requirements, were excluded from the analysis. The enrollment period for GRLS occurred between September 2012 and March 2015 and the second visit for each participant occurred between September 2013 and March 2016.

### 2.3 Data collection and analysis

Data collected at the time of enrollment in GRLS were used for this study. Most variables were analyzed based on individual survey questions (eg, sex, age), but individual behavior‐related questions were combined to form composite scores for behavior variables (eg, owner‐directed aggression). Composite scores were calculated using a method outlined in the Canine Behavioral Assessment and Research Questionnaire (C‐BARQ).^11^, ^12^, ^13^ Lower composite scores were favorable for all behavior variables except trainability. Categorical variables were collapsed to facilitate analysis. Composite behavior scores, activity level, walk frequency, and aerobic activity frequency were dichotomized and sleeping location was categorized (Table 1).

**TABLE 1:** Characteristics of the dogs enrolled in the Golden Retriever Lifetime Study at enrollment (N = 3035), in full compliance with study requirements at the end of year 1 (N = 2626), and noncompliant at the end of year 1 (N = 192). Total values for each category are followed by percent of total within each cell (n, [%])

Characteristic	Category	All dogs (n = 3035)	Year 1 total compliance (n = 2626)	Year 1 owner noncompliant (n = 192)	Univariable *P* value
Sex^a^					.49
	Male intact	1028 (33.9)	882 (33.6)	68 (35.4)	
	Female intact	874 (28.8)	745 (28.4)	55 (28.7)	
	Male neutered	507 (16.7)	453 (17.3)	25 (13.0)	
	Female spayed	626 (20.6)	546 (20.8)	44 (22.9)	
Age (months)					<.01
	<12	1034 (34.1)	926 (35.3)	48 (25.0)	
	12‐23	1413 (46.6)	1211 (46.1)	93 (48.4)	
	24‐35	579 (19.1)	485 (18.5)	46 (24.0)	
	36‐48	9 (0.3)	4 (0.2)	5 (2.6)	
Geographic region					.09
	South	721 (23.8)	632 (24.1)	38 (19.8)	
	Mountain	412 (13.6)	360 (13.7)	22 (11.5)	
	Northeast	636 (21.0)	568 (21.6)	38 (19.8)	
	Pacific	428 (14.1)	353 (13.4)	40 (20.8)	
	Midwest	838(27.6)	713 (27.2)	54 (28.1)	
Health insurance					.21
	Yes	577 (19.0)	509 (19.4)	30 (15.6)	
	No	2458 (81.0)	2117 (80.6)	162 (84.4)	
Clinic AAHA status					.11
	Member, accredited	786 (25.9)	675 (25.7)	52 (27.2)	
	Member, non‐accredited	318 (10.5)	287 (10.9)	12 (6.3)	
	Not a member	1931 (63.6)	1664 (63.4)	128 (66.7)	
Dogs in study per vet					.06
	1–5	2779 (91.6)	2390 (91.0)	183 (95.3)	
	6‐10	216 (7.1)	198 (7.5)	9 (4.7)	
	>11	40 (1.3)	38 (1.5)	0 (0)	
Activity level					.04
	Very active	1542 (50.8)	1329 (35.5)	111 (57.8)	
	Less active	1493 (49.2)	1297 (49.4)	81 (42.2)	
Primary activity					.05
	Companion/pet	2519 (83.0)	2187 (83.3)	152 (79.2)	
	Obedience	83 (2.7)	75 (2.9)	4(2.1)	
	Show	134 (4.4)	103 (3.9)	16 (8.3)	
	Breeding	43 (1.4)	37 (1.4)	2 (1.0)	
	Agility	105 (3.5)	100 (3.8)	3 (1.6)	
	Hunting	41 (1.4)	32 (1.2)	5 (2.6)	
	Field trials	58 (1.9)	47 (1.8)	7 (3.7)	
	Search and rescue	12 (0.4)	12 (0.5)	0 (0)	
	Service dog	35 (1.2)	29 (1.1)	3 (1.6)	
	Other	5 (0.2)	4 (0.2)	0 (0)	
Spends most time					.01
	Indoors	2041 (67.3)	1786 (68.0)	110 (67.3)	
	Outdoors	127 (4.2)	104 (4.0)	9 (4.2)	
	Both	867 (28.6)	736 (28.0)	73 (28.6)	
Sleeps at night					<.01
	In bedroom on bed	634 (20.9)	559 (21.3)	32 (16.7)	
	In bedroom elsewhere	1607 (53.0)	1409 (53.7)	85 (44.3)	
	Elsewhere in house	719 (23.7)	602 (22.9)	64 (33.3)	
	Garage	22 (0.7)	13 (0.5)	5 (2.6)	
	Outside (kennel, run, yard)	53 (1.8)	43 (1.6)	6 (3.1)	
Travel					.24
	Yes	476 (15.7)	403 (15.4)	36 (18.8)	
	No	2559 (84.3)	2223 (84.7)	156 (81.3)	
Leash walk frequency					.02
	At least daily	1847 (60.9)	1628 (62.0)	101 (52.6)	
	Less than daily	1188 (39.1)	998 (38.0)	91 (47.4)	
Professional grooming					.04
	>once/month	269 (8.9)	227 (8.6)	28 (14.6)	
	2–4 times/year	875 (28.8)	765 (29.1)	54 (28.8)	
	Yearly	83 (2.7)	72 (2.7)	7 (3.7)	
	Never	1808 (59.6)	1562 (59.5)	103 (53.7)	
Home grooming					.02
	>once/month	1628 (53.6)	1394 (53.1)	110 (53.6)	
	2–4 times/year	1147 (37.8)	1009 (38.4)	58 (37.8)	
	Yearly	35 (1.2)	33 (1.3)	1 (1.2)	
	Never	225 (7.4)	190 (7.2)	23 (7.4)	
Heartworm prevention‐all year					.20
	Yes	2088 (68.8)	1815 (69.1)	124 (68.8)	
	No	947 (31.2)	811 (30.9)	68 (31.2)	
Flea/tick prevention‐all year					.18
	Yes	595 (19.6)	507 (19.3)	45 (23.4)	
	No	2440 (80.4)	2119 (80.7)	147 (76.6)	
Rabies 1 year vaccine					.24
	Yes	1659 (54.7)	1432 (54.5)	95 (50.5)	
	No	1376 (45.3)	1194 (45.5)	97 (49.5)	
All vaccines missing					.01
	Yes	300 (9.9)	250 (9.5)	30 (15.6)	
	No	2735 (90.1)	2376 (90.5)	162 (84.4)	
Owner‐directed aggression					.07
	0–1	2933 (96.6)	2536 (96.6)	188 (97.9)	
	>1	62 (2.0)	56 (2.1)	1 (0.5)	
Chasing behavior					0.22
	0–1	94 (3.1)	78 (3.0)	9 (4.7)	
	>1	2864 (94.4)	2479 (94.4)	179 (93.2)	
Excitability					0.01
	0–1	807 (26.6)	710 (27.0)	37 (19.3)	
	>1	2208 (72.8)	1897 (72.2)	155 (80.7)	

^a^ Not included in multivariable model due to P‐value.

Variables included in the modeling approach for owner noncompliance were the dog's sex and age at enrollment, geographical region of residence (Northeast, Midwest, Mountain, South, Pacific), dog insurance (yes, no), clinic American Animal Hospital Association (AAHA) status (AAHA member at accredited hospital, AAHA member at nonaccredited hospital, not an AAHA member), number of study dogs enrolled with the same veterinarian, number of study dogs with the same owner, where dog was acquired (breeder, shelter/rescue, pet store, friend/relative or neighbor, via internet or newspaper, other), activity level (very active, less active), primary activity (companion, obedience, show, breeding, agility, hunting, field trials, search and rescue, service, other), where dog spends most time (inside, outside, both), where dog sleeps at night (bedroom on bed, bedroom elsewhere, elsewhere in house, garage, outside), home type (single family, apartment/condo/townhome), travel frequency (assessing if owner traveled with dog for 2 weeks or more within previous 12 months), leash walk frequency (daily or less than daily), aerobic activity frequency (daily or less than daily), professional grooming frequency (more than once per month, 2‐4 times per year, yearly, never), home bathing and/or grooming frequency (more than once per month, 2‐4 times per year, yearly, never), heartworm prevention frequency (assessing if used all year, seasonally, or not at all), flea/tick prevention frequency (assessing if used all year, seasonally, or not at all), rabies 1 year vaccine given in past 12 months (yes, no), rabies 3 year vaccine given in past 12 months (yes, no), no vaccines given (yes, no), and the composite scores for stranger‐directed aggression, owner‐directed aggression, stranger‐directed fear, nonsocial fear, dog‐directed fear and aggression, separation anxiety, attachment behavior, trainability, chasing behavior, excitability, and pain sensitivity.

A logistic regression model was constructed using a backwards, stepwise procedure with commercially available software in order to estimate the odds ratios using owner noncompliance as the outcome of interest (StataCorp. 2013, Stata Statistical Software: Release 13, College Station, TX: StataCorp LP). Univariable logistic regression models were used to screen individual exposure variables and a wide statistical association with the outcome of noncompliance (*P* ≤ .25) was required for inclusion in the multivariable model. The final multivariable logistic regression model was constructed using a critical *α* for retention ≤0.05. Excluded variables were reintroduced to the final model to evaluate confounding effects (identified by ≥20% change in parameter estimates). First‐order interaction terms for variables included in the final multivariable model were evaluated. The Pearson chi‐square goodness‐of‐fit test was used to evaluate model fit. Odds ratio (OR) and 95% confidence intervals (95% CI) were calculated using the final multivariable logistic regression model.

## 3 RESULTS

### 3.1 Description of population

The initial GRLS population consisted of 3044 dogs. Nine dogs died during year 1 and were removed from the data set. Thus, there were 3035 dog owners included in this study (Table 1). Of these owners, 2626 (86.5%) dogs were fully compliant at the end of year 1 and 192 (6.3%) dogs were noncompliant. The dog population was approximately evenly distributed between male (50.6%) and female (49.4%), with the majority of dogs sexually intact (62.7%) at the time of enrollment. Dogs ranged in age from 5 to 43 months at the time of enrollment, and 80.7% were under the age of 24 months. The study population was approximately evenly distributed across the 5 regions of the continental United States (Table 1).

### 3.2 Logistic regression model

Of the 36 variables analyzed using univariable logistic regression models, 20 exposure variables were included in the initial multivariable model: dog age, region, dog insurance, clinic AAHA status, number of dogs in study under veterinarian, activity level, primary activity, where dog spends most time, where dog sleeps at night, travel frequency, leash walk frequency, professional grooming, home grooming, all year heartworm prevention, all year flea/tick prevention, rabies 1 year vaccine, no vaccines given, owner‐directed aggression, chasing behavior, and excitability (Table 1).

Variables remaining in the final multivariable model were age at enrollment, where dog spends most time, where dog sleeps at night, home grooming frequency, no vaccines given, and excitability (Table 2). No confounding variables were detected. There was 1 significant interaction term between the variables pertaining to home grooming and/or bathing and vaccination status which was included in the final model. Without the interaction term, home grooming *P* = .012 [2‐4 times per year (OR: 0.71, 95% CI: 0.51, 0.99), yearly (OR: 0.36, 95% CI: 0.05, 2.75), never (OR: 1.59, 95% CI: 0.98, 2.58), >monthly (reference)] and unvaccinated *P* = .031 (OR: 1.59, 95% CI: 1.04, 2.41). With the interaction term, home grooming *P* = .005 [2‐4 times per year (OR: 0.77, 95% CI: 0.55, 1.09), yearly (OR: 0.41, 95% CI: 0.05, 3.16), never (OR: 1.93, 95% CI: 1.17, 3.19), >monthly (reference)] and unvaccinated *P* = .0041 (OR: 3.72, 95% CI: 1.51, 9.18). Although not included in the final model, the number of dogs enrolled in GRLS with the same veterinarian approached statistical significance (*P* = .051, OR: 0.92, 95% CI: 0.85, 1.00). The Pearson chi‐square statistic indicated that the model fit the data sufficiently well (*X* ^2^ = 984.62; *P*‐value = .40).

**TABLE 2:** Final multivariable logistic regression model with the outcome of owner noncompliance at the end of year 1 of the Golden Retriever Lifetime Study for all dogs enrolled (N = 3035)

Characteristic	Category	OR	95% CI		*P* value
Age (months)	Continuous	1.05	1.03	1.07	<.01
Spends most time	Outdoors	0.82	0.36	1.85	.03
	Both	1.50	1.09	2.05	
	Indoors	Reference			
					
Sleeps	Garage	5.66	1.89	17	<.01
	Outside	2.31	0.85	6.29	
	Elsewhere in house	1.85	1.31	2.60	
	Bedroom, on bed	0.90	0.59	1.37	
	Bedroom, elsewhere	Reference			
					
Home grooming^a^	2–4 times per year	0.77	0.55	1.09	<.01
	Yearly	0.41	0.05	3.16	
	Never	1.93	1.17	3.19	
	At least monthly	Reference			
					
No vaccines^a^	Yes	3.72	1.51	9.18	<.01
	No	Reference			
					
Excitability	>1	1.56	1.07	2.26	.02
	0–1	Reference			

^a^ Interaction, adjusted values.

In the adjusted model, owners of dogs who sleep anywhere other than the owner's bedroom at night had higher odds of being noncompliant at their second study visit than were owners who allowed their dogs to sleep in their bedroom. Owners of dogs who sleep in the garage had nearly 6 times higher odds of being noncompliant (OR: 5.66, 95% CI: 1.89, 16.96) compared to owners of dogs who sleep in the bedroom. Additionally, owners of dogs that are never bathed and/or groomed at home had almost 2 times higher odds of being noncompliant (OR:1.93, 95% CI: 1.17, 3.19) than owners of dogs who are groomed at home at least once a month. Owners of dogs without record of vaccination had nearly 4 times higher odds of being noncompliant with study protocols (OR: 3.72, 95% CI: 1.51, 9.18) than owners of vaccinated dogs. Owners had slightly higher odds of being noncompliant (OR: 1.05, 95% CI: 1.03, 1.07) with each additional month added to their dog's age at the time of enrollment in GRLS. Owners of dogs who spend most of their time both inside and outside had 1.5 times higher odds of being noncompliant (OR: 1.50, 95% CI: 1.09, 2.05) than owners of dogs who spend most time indoors. Finally, owners of dogs who were more excitable (composite score > 1) had nearly 2 times higher odds of being noncompliant (OR: 1.56, 95% CI: 1.07, 2.26) than owners of dogs who were less excitable (composite score 0‐1).

## 4 DISCUSSION

Out of an extensive baseline survey, 6 variables were statistically significant predictors of owner noncompliance by the end of year 1 after enrollment of GRLS. These predictor variables are simple questions that might help guide future efforts for recruiting dog owners for other cohort studies by predicting owner compliance with study requirements. The pool of owners of potential participants that is predicted to be more likely to be noncompliant based off of screening questions such as “Where does your dog sleep at night?” could either be excluded or oversampled in order to maximize data collection. Oversampling owners predicted to be noncompliant would potentially avoid bias that would be introduced by simply excluding potentially noncompliant owners altogether.

Another strategy that could increase owner compliance is the use of incentives, such as rewards or gifts, being given at the completion of the annual survey and veterinary visit or prepaid cash incentives sent with the reminder in advance of the due date for the annual survey. However, the use of such incentives in longitudinal studies has been shown to be variably effective and can disproportionately attract different types of respondents.^14^, ^15^ In addition, the use of incentives with large study populations can be cost prohibitive.

The survey question with the largest OR for owner compliance was where an owner allows their dog to sleep at night. The answer to this question might be a surrogate for questions about how the owner perceives the relationship between themselves and their dog. An association has already been established between the strength of an owner's bond with their pet and an increased likelihood to follow veterinarian recommendations and to seek preventive care for their pet.^16^ Perhaps the strength of an owner's bond with their pet is also related to where the owner is likely to allow their dog to sleep and thus indirectly is associated with compliance with study protocols. Therefore, questions relating to an owner's bond with their dog might help predict owner compliance in a cohort study such as GRLS and other clinical studies that require a high level of owner engagement. The location where a dog spends most of its time and home grooming frequency, both statistically significant indicators of owner compliance, could give similar information regarding an owner's bond with their dog as does the location where a dog sleeps at night.

Vaccination status was another statistically significant indicator of owner compliance, which might indicate that the level of preventive veterinary care a dog receives, is associated with owner study compliance. Conversely, a dog's level of excitability might have an impact on an owner's likelihood of taking their dog to the veterinarian as owners of dogs who are more excitable might not be comfortable or willing to take their dog to the veterinarian as compared to owners of dogs who are less excitable. Additionally, although all dogs were relatively young at enrollment, perhaps owners of the youngest dogs were better able to establish GRLS compliance as an integral part of their care regimen when their dog was still a puppy. The greater the number of dogs in the study cared for by the same veterinarian approached statistical significance. It is possible that veterinarians with more dogs involved in GRLS positively influenced owners to be more compliant through year 1, but it is also possible that highly motivated owners (who might be more likely to be compliant through year 1) are more likely to go to a high‐quality and engaged veterinarian who cares for more dogs in GRLS. The practical significance of this factor could become clearer as the study progresses.

An important limitation to this study is the potential for information bias introduced by having the owner answer lifestyle and behavior questions about their own dog. Not only can dog owners view their dog's behavior with bias due to their close relationship with the dog, but dog owners might also be impacted by social desirability bias, tending to answer survey questions in a way that they believe might be viewed more favorably by others.^17^, ^18^ There could also be selection bias involved since study participation was voluntary; therefore, data were only provided by owners who chose to share information about their dog's lifestyle. However, since the population is distributed throughout the United States, data might be considered representative of Golden Retriever owners throughout the country. Additionally, Golden Retrievers were the only breed involved in GRLS, so all data were specific to Golden Retrievers. Thus, some variables such as home grooming could have different compliance impacts on owners of breeds of different activity levels, size, and coat type. It is not known if the risk factors associated with compliance at the end of the first year of GRLS will affect owner compliance past year 1. Ideally, owner compliance through year 1 would be a reliable predictor for owner compliance throughout the length of the entire cohort study, but it is possible that factors affecting owner compliance will change over the course of the study and with their dog's increasing age. This study's results might be most helpful for predicting owner compliance in cohort studies lasting approximately 1 year. Analyzing GRLS owner compliance again after several years will be important to understanding what factors affect owner compliance in cohort studies lasting up to 15 years.

## CONFLICT OF INTEREST DECLARATION

Authors declare no conflict of interest.

## OFF‐LABEL ANTIMICROBIAL DECLARATION

Authors declare no off‐label use of antimicrobials.

## INSTITUTIONAL ANIMAL CARE AND USE COMMITTEE (IACUC) OR OTHER APPROVAL DECLARATION

Authors declare no IACUC or other approval was needed.

## HUMAN ETHICS APPROVAL DECLARATION

Authors declare human ethics approval was not needed for this study.
